# Definition, Frequency and Risk Factors for Intra-Operative Spinal Cord Injury: A Knowledge Synthesis

**DOI:** 10.1177/21925682231190613

**Published:** 2024-03-25

**Authors:** Michael G. Fehlings, Ayesha Quddusi, Andrea C. Skelly, Erika D. Brodt, Ali Moghaddamjou, Anahita Malvea, Nader Hejrati, Nisaharan Srikandarajah, Mohammed Ali Alvi, Shay Stabler-Morris, Joseph R. Dettori, Lindsay A. Tetreault, Nathan Evaniew, Brian K. Kwon

**Affiliations:** 1 Division of Neurosurgery and Spine Program, Department of Surgery, 7938 University of Toronto, Toronto, ON, Canada; 2 Division of Neurosurgery, Krembil Neuroscience Centre, Toronto Western Hospital, 7989 University Health Network, Toronto, ON, Canada; 3 Institute of Medical Science, 7938 University of Toronto, Toronto, ON, Canada; 4 Aggregate Analytics, Inc., Fircrest, WA, USA; 5 Spectrum Research, Inc., Steilacoom, WA, USA; 6 Department of Neurology, 12297 NYU Langone Medical Center, New York, NY, USA; 7 McCaig Institute for Bone and Joint Health, Department of Surgery, Orthopaedic Surgery, Cumming School of Medicine, 2129 University of Calgary, AB, Canada; 8 Department of Orthopaedics, 8166 University of British Columbia, Vancouver, BC, Canada; 9 International Collaboration on Repair Discoveries (ICORD), 8166 University of British Columbia, Vancouver, BC, Canada

**Keywords:** spinal cord injury, neuro, trauma, intra-operative spinal cord injury

## Abstract

**Study Design:**

Mixed-methods approach.

**Objectives:**

Intra-operative spinal cord injury (ISCI) is a devastating complication of spinal surgery. Presently, a uniform definition for ISCI does not exist. Consequently, the reported frequency of ISCI and important risk factors vary in the existing literature. To address these gaps in knowledge, a mixed-methods knowledge synthesis was undertaken.

**Methods:**

A scoping review was conducted to review the definitions used for ISCI and to ascertain the frequency of ISCI. The definition of ISCI underwent formal review, revision and voting by the Guidelines Development Group (GDG). A systematic review of the literature was conducted to determine the risk factors for ISCI. Based on this systematic review and GDG input, a table was created to summarize the factors deemed to increase the risk for ISCI. All reviews were done according to PRISMA standards and were registered on PROSPERO.

**Results:**

The frequency of ISCI ranged from 0 to 61%. Older age, male sex, cardiovascular disease including hypertension, severe myelopathy, blood loss, requirement for osteotomy, coronal deformity angular ratio, and curve magnitude were associated with an increased risk of ISCI. Better pre-operative neurological status and use of intra-operative neuromonitoring (IONM) were associated with a decreased risk of ISCI. The risk factors for ISCI included a rigid thoracic curve with high deformity angular ratio, revision congenital deformity with significant cord compression and myelopathy, extrinsic intradural or extradural lesions with cord compression and myelopathy, intramedullary spinal cord tumor, unstable spine fractures (bilateral facet dislocation and disc herniation), extension distraction injury with ankylosing spondylitis, ossification of posterior longitudinal ligament (OPLL) with severe cord compression, and moderate to severe myelopathy.

**Conclusions:**

ISCI has been defined as “a new or worsening neurological deficit attributable to spinal cord dysfunction during spine surgery that is diagnosed intra-operatively via neurophysiologic monitoring or by an intraoperative wake-up test, or immediately post-operatively based on clinical assessment”. This paper defines clinical and imaging factors which increase the risk for ISCI and that could assist clinicians in decision making.

## Introduction

Intra-operative spinal cord injury (ISCI) is one of the most feared and devastating complications of spine surgery.^1,2^ To date, studies have described ISCI using various definitions and diagnostic criteria. Therefore, the reported frequency and risk factors of ISCI have also varied in the literature. Part of this variability in definition is due to differences in the methods used to determine and define ISCI. Specifically, some studies use findings of intra-operative neuromonitoring (IONM) to define ISCI, while others rely only on post-operative neurological examination findings. The lack of a standardized definition and diagnostic criteria for ISCI is a major challenge in finding solutions to minimize ISCI. Unifying nomenclature and developing standardized diagnostic criteria are essential for accurately quantifying the frequency of ISCI and determining the risk factors associated with it. To the authors’ knowledge, there has been no systematic synthesis of the literature to date that defines ISCI, examines the frequency and outcomes of ISCI, describes the role of IONM, and/or reviews the management strategies in the case of ISCI (both with and without the use of IONM). Additionally, the factors that heighten the risk for sustaining an ISCI have been poorly characterized.

To address these gaps in knowledge, a mixed-methods knowledge synthesis was undertaken. To begin, a formal systematic review was planned and the protocol was registered on PROSPERO (CRD42022298841). The original contextual and key questions as well as the PICOTS (P = Population, I = Intervention, C = Comparators, O = Outcomes, T = Timing, S = Study Design) are found in Table 1. Briefly, the proposed review intended to,

(i) Provide context regarding case definitions and diagnostic criteria of ISCI, as well as the use and accuracy of IONM.
(ii) Evaluate the risk factors for the development of ISCI.
(iii) Address key questions related to comparative effectiveness and harms of ISCI management options.

**Table 1.** Review Key Question on Risk factors for ISCI: Inclusion and exclusion criteria - population, prognostic factors, outcomes, studies.

	Inclusion	Exclusion
Patients	• adolescents (≥11 years to <18 years old) or adults (≥18 years) undergoing any type of spine surgery for any indication or spine-related pathology (including trauma-related pathology, conus injuries, cauda equina injuries)	• patients <11 years old • patients undergoing surgery for pathologies not involving the spine (eg, vascular surgeries) • patients with new post-operative compression (eg, hematoma, abscess) • patients with root-level deficits • patients with neurological deficits due to cranial pathology (eg, stroke)
Prognostic factors of interest	**Primary factors of interest** • **clinical/pathology factors** (eg, tumors [intra or extra-dural], scoliosis or other spinal deformity, myelopathy, trauma) • **surgical factors** (eg, surgical procedure, surgical approach, number of levels) **Potential confounding factors** • **demographic factors** (age, sex, BMI, smoking)	
Outcome	Documented intra-operative spinal cord injury defined as: new or worsening neurological deficit attributable to spinal cord dysfunction that occurs as the result of spine surgery either intra-operatively (diagnosed via intra-operative monitoring) or in the immediate post-operative period (based on clinical assessment); includes cauda-equina syndrome and conus compression in addition to cord compression	• root-level injuries, nerve palsies, peripheral nerve injury • hematoma, abscess • ischemia following aorta or vascular surgery • anesthesia effects • Brain/intracranial effects, pathologies (eg, stroke)
Studies	Studies with the highest methodological quality were focused on • prospective studies: If no prospective studies were available, retrospective studies were considered • only studies which controlled for confounding (eg, patient, or clinical factors) were included for formal risk factor evaluation • case series and narrative reviews were considered in the absence of formal controlled studies of risk factors	• studies with <15 patients • for formal risk factor evaluation, studies which do not control for potential confounding • case reports, case series, conference proceedings, abstracts, letters, white papers, cross-sectional studies • Animal or cadaver studies

Broad scoping literature searches of published literature were conducted, along with input from experts from the Guidelines Development Group (GDG), that yielded substantial yet limited evidence to perform a full systematic review, except for the question regarding risk factors and accuracy of IONM. Given this, our original plan was revised to incorporate scoping reviews that addressed (1) definitions, frequency, and risk factors for ISCI, (2) the use and accuracy of IONM for diagnosis of ISCI, and (3) reported management approaches for ISCI and related harms.

The purpose of this study was to conduct a mixed-methods knowledge synthesis of the literature to address the following questions:

### Contextual Question

What definitions and/or monitoring thresholds are used to define ISCI, and what is the reported frequency of ISCI in the existing literature?

### Key Question

What are the high-risk factors for ISCI?

## Methods

A scoping review was conducted to address the contextual question on the definition and frequency of ISCI. A systematic review was conducted to address the key question examining risk factors for ISCI. Evidence gathered from these underwent review and discussion by the GDG using the Delphi process. Methods used for the systematic review of ISCI risk factors were in accordance with the Agency for Healthcare Research and Quality’s (AHRQ) Methods Guide for Effectiveness and Comparative Effectiveness Review.^3^ The contextual question was answered based on the U.S. Preventive Services Task Force (USPSTF) methods^4^ for contextual questions, and based on the citations identified via formal literature search as well as additional gray literature.

### Study Design

The study was designed with a mixed-methods approach that incorporated evidence from a scoping review and a systematic review, as well as input from a GDG of experts on the topic.

### Literature Search Strategies

#### Literature Databases

**Figure 1.**
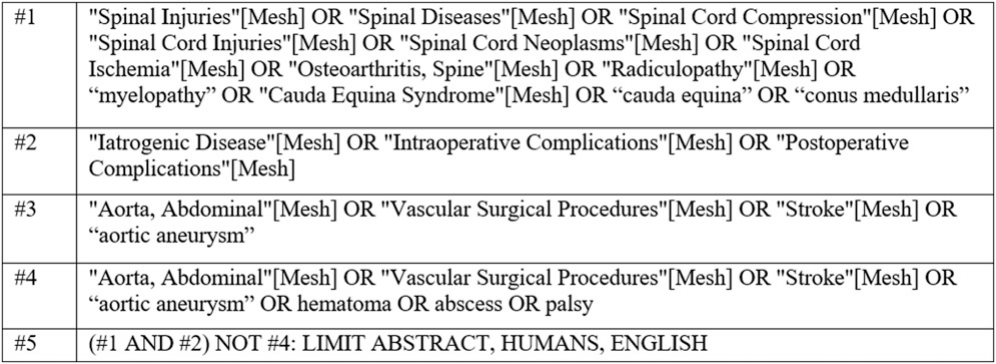
Search Strategy.

The Cochrane Library and MEDLINE^®^ were extensively searched. Included studies were limited to publications in English. Search terms that were used are summarized in Figure 1. Citations from the search were deduplicated and dual-screened for inclusion. In addition, sources of gray literature were reviewed, including professional society guidelines, select pertinent book chapters, and other similar literature, primarily for the contextual question on the definition and frequency of ISCI. Citations suggested by the GDG were compared against the criteria for inclusion and exclusion. The EndNote library was searched using Key Words “risk”.

#### Publication Date Range

The search included citations from database inception to January 26, 2022.

#### Hand Searching

Reference lists of included studies, systematic reviews, and pertinent gray literature were also evaluated for relevant studies.

### Process for Selecting Studies

For the contextual question on the definition and frequency of ISCI ***,*** studies reporting on thresholds used to identify ISCI were selected if they reported on a minimum of three patients and satisfied the population inclusion/exclusion criteria in Table 1.

Consistent with other reviews that support guideline updates, studies with the least potential for bias using a “best evidence” approach were focused on. Randomized control trials (RCTs) and high-quality prospective comparative cohort studies that controlled for confounding factors were included as the primary evidence source. In the absence of high-quality studies, lower-quality studies (eg case series) and narrative reviews were also considered.

For the systematic review on risk factors of ISCI, the pre-established criteria summarized in Table 1 were used to screen citations (titles and abstracts) identified by the literature search. Any citation deemed not relevant for full-text review was reviewed by a second researcher to assure accuracy and completeness. Potentially eligible citations identified for inclusion by at least one of the reviewers were retrieved for full-text screening. Each full-text article was independently reviewed for eligibility by two team members. Any disagreements were resolved by consensus. Studies excluded after full-text review with reasons for exclusion are listed in Appendix A.

### Data Abstraction and Management

To address the contextual question on definition and frequency, the information on IONM definitions or thresholds and the resolution or persistence of neurological deficit was noted from the selected studies. Data was collected on how the included studies determined there was a neurological deficit. Neurological deficit is determined using clinical neurological assessment and categorizing the degree of deficit based on a grading system. Commonly used grading systems in research and clinical practice for categorizing the severity of a neurological deficit due to spinal cord injury are the American Spinal Injury Association (ASIA) Impairment Scale (AIS) grading system, McCormick grade, and Medical Research Council (MRC) scale for Muscle Strength grading system. Neurological deficit can also be detected by changes in IONM parameters. These include somatosensory evoked potentials (SSEPs) that monitor the integrity of the dorsal column-medial lemniscal pathway, motor evoked potentials (MEPs) and transcranial motor evoked potentials (TcMEPs) that monitor the integrity of the corticospinal tracts, and electromyography (EMG) that indicates the integrity of individual nerves.^5^ Importantly, clinical examination and IONM are not mutually exclusive when reporting on ISCI and studies can use either or both methods to document ISCI.

After studies were selected for inclusion in the systematic review on risk factors, standardized data abstraction included the following (at minimum): patient characteristics (age, sex, comorbidities), assessment and categorization of injury severity (AIS) and level of spinal cord injury, indication for spine surgery (eg, scoliosis, tumor), clinical/disease characteristics (eg, myelopathy), surgical factors (eg approach, levels, instrumentation), adjunctive treatments (eg, steroids, vasopressors), and study-related characteristics (eg, sample size, design, control of confounding, the timing of follow-up).

### Assessment of Methodological Risk of Bias of Individual Studies

The studies that described ISCI frequency were small case series and were not critically appraised. All were of poor quality.

Pre-defined criteria were used to assess the risk of bias of included non-randomized studies using the Quality in Prognosis Studies (QUIPS) tool for studies evaluating risk factors.^6^ Two methodologists independently assessed the risk of bias. Disagreements were resolved by a discussion leading to consensus. Based on the risk of bias assessment, studies were rated as “good,” “fair” or “poor” quality based on the criteria in Table 2.

**Table 2.** Criteria for grading the quality of individual studies.

Rating	Description and Criteria
**Good**	• low risk of bias, most criteria for quality are met, and results are generally considered valid • Valid methods for selection, inclusion, and treatment allocation; report similar baseline characteristics in different treatment groups; clearly describe attrition and have low attrition; appropriate means for preventing bias and use of appropriate analytic methods
**Fair**	• some study flaws: may not meet all criteria for good quality, but no flaw is likely to cause major bias that would invalidate results; the study may be missing some information making it difficult to assess limitations and potential problems. This is a broad category; results from studies may or may not be valid
**Poor**	• significant flaws that imply biases of various kinds that may invalidate results; most criteria for a good quality study are not met and/or “fatal flaws” in design, analysis, or reporting are present; large amounts of missing information; discrepancies in reporting; or serious problems with intervention delivery

### Data Synthesis

The data was qualitatively summarized in tables using ranges, descriptive analyses, and interpretation of the results. Data on the frequency of ISCI were qualitatively synthesized.

Adjusted odds ratios provided by authors were reported. Clinical and methodological heterogeneity across studies precluded the pooling of studies.

### Grading the Strength of Evidence for Major Comparisons and Outcomes

The overall quality and strength of evidence (SOE) was assessed based on the application of Grading of Recommendations, Assessment, Development, and Evaluations (GRADE), described in the AHRQ Methods Guide.^3^ GRADE guidance related to synthesis of risk factors was used.^7,8^ The SOE was assigned an overall grade of high, moderate, low, or very low. SOE was evaluated by one methodologist and then reviewed independently by a second for consistency and validity before the final assessment. Disagreements were resolved by consensus. For the systematic review of risk factors, studies were initially considered to be a high quality of evidence. The evidence was downgraded based on the aggregate assessment of risk of bias across studies reporting on the outcome, consistency, imprecision, directness, and publication bias. Strength of evidence was not applied to the results for the contextual question.

## Results

### Contextual Question

What definitions and/or monitoring thresholds are used to define ISCI, and what is the reported frequency of ISCI in the existing literature?

### Definition of Intra-operative Spinal Cord Injury

Studies provided variable definitions and thresholds for diagnosis of ISCI, which are reported in Table 3.

**Table 3.** Contextual Question: Criteria/thresholds for intra-operative SCI and frequency.

Deformity				
Author, year	Definition	Indication of surgery	Alert Definition/Threshold	Timepoint
Kato 2018 (SCOLI)	Loss of LEMS at discharge in comparison to preoperative (baseline) status	Complex adult spinal deformity	Major decline: LEMS loss of 5 points or more Minor decline: LEMS loss of less than 5 points	LEMS compared to preoperative baseline
Fehlings 2018 (SCOLI)	Postoperative deterioration in ASIA LEMS compared with preoperative status	Complex adult spinal deformity	Postoperative deterioration in ASIA LEMS compared with preoperative status, threshold NR	ASIA LEMS at discharge
Lenke 2016 (SCOLI)	Decrease in ASIA LEMS postoperative compared to preoperative	Complex adult spinal deformity	Decrease in LEMS postop compared to preoperative, with normal LEMS = 50, abnormal LEMS <50	ASIA LEMS at 6 weeks
Tumors				
Harel 2017	Decrease in McCormick grade	Intradural extramedullary tumors	Mean McCormick grade. Patients graded as either stable or deteriorated compared to preoperative state	Immediate postoperative, further follow-up not defined
Kang 2017	Motor power as graded by the Medical research Council system	Intradural extramedullary and epidural metastatic spinal tumors	Postoperative drop in patient’s motor power by ≥ 1 grade	Acute period, 2 days postoperative, and before rehabilitation treatment
Korn 2014	Decrease in modified McCormick grade	Extramedullary spinal cord tumors	Mean McCormick grade. Patients graded as either stable or deteriorated compared to preoperative state	3 hours postoperative, 3 months, 6 months follow-up
Lakomkin 2017	IONM signal change	Spinal cord tumors	True-positive defined when IONM signal change correlated either with a new, permanent postoperative motor and/or sensory deficit that remained present at a 6months follow-up	6 months
Sala 2006	Decrease in McCormick grade	Intramedullary spinal cord tumor	Patients given a value of 0 if they remained stable after surgery, and -1, -2, or -3 if they deteriorated after surgery by 1, 2, or 3 grade(s) on the McCormick scale, and +1, +2, +3 if they improved	Discharge and 3+ month follow-up
Skinner 2005	Intra-operative warning to surgeon	Intramedullary spinal cord lesions	True-positive case defined as a new or worsened postoperative motor deficit that was predicted by means of an intra-operative warning to the surgeon	Postoperative, long-term follow-up varied by patient (range: 1-28 months)

The search generated several studies which were grouped based on indication for surgery into those on deformity surgery and those on tumor surgery. As seen in Table 3, the definitions in the deformity (Kato 2018,^10^ Fehlings 2018,^11^ Lenke 2016^12^) groups were centered on deterioration in lower extremity motor score (LEMS). LEMS in these studies was described using the ASIA grading system. In contrast, the tumor studies defined ISCI based on change in McCormick grade, MRC grading system, or IONM prompts (Harel 2017,^13^ Kang 2017,^14^ Korn 2015,^15^ Lakomin 2017,^16^ Sala 2006,^17^ Skinner 2005^18^). These studies reported the criteria for defining an ISCI as either a change compared to preoperative LEMS/ASIA score, a LEMS change of 5 points, change in McCormick grade, or in the case of neurophysiologic monitoring, a signal that correlated with a post-operative motor deficit. Briefly, these include MEP amplitude changes of more than 50-80%, SSEP amplitude changes of more than 50-60%, latency prolongation of 10% or 3 milliseconds, TcMEP amplitude change of more than 50-80%, muscle threshold of more than 100 V, EMG amplitude change of more than 50%, sustained bursts/trains, lack of waveform, or a D-wave decrease in amplitude of more than 50%.

The following definition was put forth by the GDG after reviewing evidence from the scoping review and subsequently voting and discussing it in depth, in accordance with the Delphi Process^9^:

“a new or worsening neurological deficit attributable to spinal cord dysfunction during spine surgery that is diagnosed intra-operatively via neurophysiologic monitoring, by an intraoperative wake-up test or immediately post-operatively based on clinical assessment.”

Deficits can include dysfunction attributable to injury of the spinal cord, conus medullaris, or cauda equina.

### Frequency of Intra-operative SCI

For the second part of the contextual question on the frequency of ISCI, a total of 61 studies (N = 15 376) were identified (Table 4). Criteria for IONM used to identify ISCI varied among these studies. The most common cut-off for amplitude drop in signal for SSEP and MEP was a drop of 50%. This cut off was employed by a majority of studies when sub-categorized according to indication and spinal level. As such, this was reported by 8 out of 13 studies on deformity, 11 out of 13 studies for tumor, 9 out of 16 studies with mixed pathologies, 7 out of 11 studies with cervical spine pathology, 3 out of 3 studies with thoracic spine pathology, and 1 out of 3 studies on patients with lumbar spine pathology. Some studies used an increase in latency of 10% with a more than 50% drop in SSEP or MEP to classify an IONM-based alert. Only cases of ISCI due to spine surgery were reported. Those due to post-operative compression (eg, hematoma), root-level deficits, or arising from surgeries for pathologies not involving the spine (eg, vascular surgeries) were not reported. Overall, new deficits ranged from 0 to 61%; with increasing granularity, it was found that when the studies were divided by pathology/level of surgery, tumor surgery demonstrated a wider range of frequencies of intra-operative deficits (0-61%) compared to deformity surgery (0-17.8%), while studies with mixed pathologies reported an intermediate range of 0-9.4%. The greatest prevalence of neurological deficit was found in lumbar level surgeries (0-28.5%). Deformity-related ISCI was more likely to resolve with up to 8% of deficits persisting, while 26.9% of tumor patients had persistent deficits. The sample sizes across studies varied from 5 to 2069, so percentages for ISCI should be interpreted cautiously.

**Table 4.** Frequency of deficits spanning pathology type and level of surgery.

Condition	Number of studies (Range of Sample Size)	New deficit% (n/N)	Resolved Deficit % (n/N)	Persistent Deficit% (n/N)
Deformity	13 studies (28 to 1121)	0% (0/144, 0/452, and 0/97) to 17.8% (5/28)	NR to 92% (200/217)	NR to 8% (17/217)
Tumor	13 studies (13 to 1017)	0% (0/19 and 0/68) to 61% (8/13)	34% (17/50) to 67% (681/1017)	7.4% (15/203) to 26.9% (14/52)
Level	Number of studies (range of sample size)	New deficit % (n/N)	Resolved deficit % (n/N)	Persistent deficit % (n/N)
Cervical	11 studies (52 to 1445)	.09 (1/1039) to 5.7% (10/175)	1.8% (1/57) to 100% (246/246)	NR
Thoracolumbar	2 studies (173 to 295)	.7% (2/295) to 5.8% (10/173)	NR	NR
Thoracic	3 studies (r 44 to 871)	.6% (5/871) to 13.4% (11/82)	NR to 100% (44/44)	NR
Lumbar	3 study (35 to 113)	0 (0/113) to 28.5% (10/35)	NR	NR
Mixed	16 studies (5 to 2069)	0% (0/2069) to 9.4 (6/64)	2.2% (9/408) to 6.3% (4/64)	.5% (1/176) to 4.9% (20/408)

#### Key Question

What Are the High-Risk factors for ISCI?

### Risk Factors for ISCI

**Figure 2.**
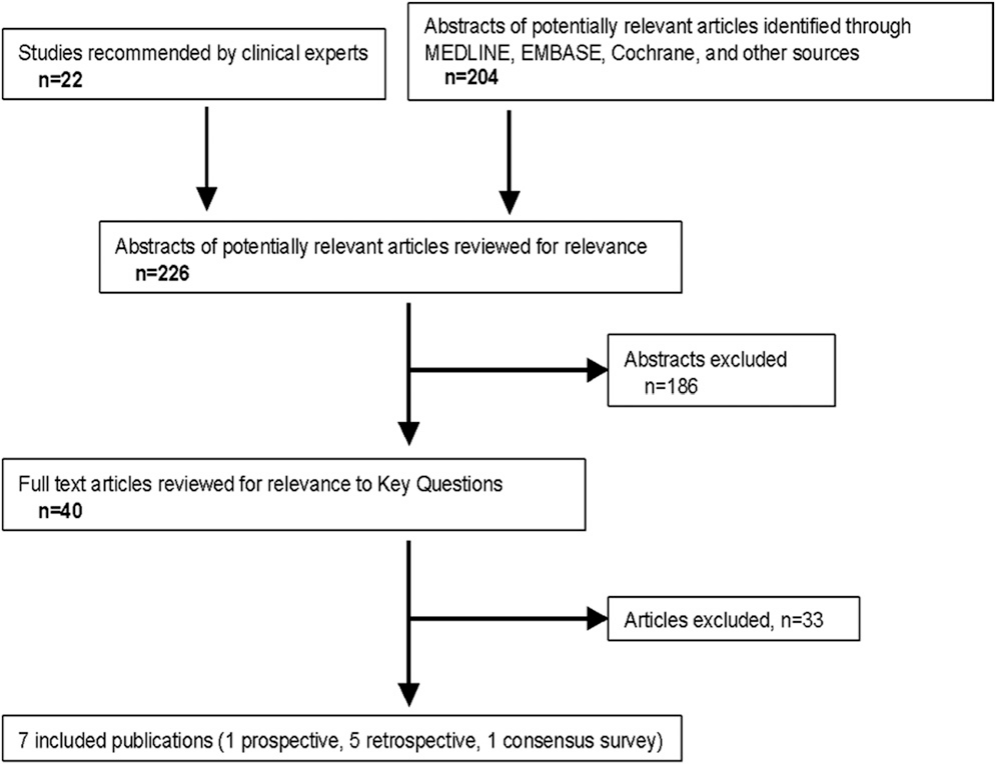
Literature search and study selection flow diagram for the systematic review on risk factors of ISCI.

From the 226 citations identified via literature search, a total of six studies were identified that provided data regarding the frequency of ISCI and conducted multivariable analysis, including four studies (Fehlings 2018,^11^ Chen 2012,^19^ Kim 2021,^20^ Romero-Munoz 2019^21^) that were recommended by clinical experts (Figure 2). An additional surgeon consensus survey (Iyer 2022)^22^ was also considered in the literature analysis, as recommended by the GDG. Data abstraction from these studies is summarized in Tables 5 and 6.

**Table 5.** Data abstraction for included studies looking at risk factors for intraoperative neurological deficits.

AuthorDesign	Population	Exclusion Criteria	Surgery	Intraoperative SCI definition and Incidence	Risk Factors Assessed^a^ Effect Estimate (95% CI)^b^	Funding COI
Deformity						
Fehlings 2018 Prospective cohort Study quality: Good	Surgical indication: ASD with an apex of the major deformity in the cervicothoracic or the thoracolumbar region between C7 and L2 inclusive undergoing complex ASD surgery^c^ Eligible: N = 272 Analyzed: N = 265 Mean age: 56.8 ± 15.4 years Males: 32%	• Substance dependency • Psychosocial disturbance • Active malignancy • Active bacterial infection • Recent history of significant spinal trauma or malignancy • Complete long-term paraplegia • Pregnancy • Prisoners • Institutionalized individuals	• Three-column osteotomy between C7 and L5 inclusive (76%) • Corrective osteotomies for revision of spinal deformity (61%) or for congenital spinal deformity (5%) • Corrective surgery for curvature with major Cobb angle of ≥80° in the coronal or sagittal plane (29%) • Reconstruction for deformity-related myelopathy (5%) • Deformity reconstruction with concomitant spinal cord decompression for OLF or OPLL (2%) • Osteotomy level Lumbar: 73% Thoracic: 43% • Surgical approach Posterior only: 77% Anterior-posterior: 23%	Decline in ASIA lower extremity motor score at discharge compared to preoperative status Incidence: 23% (61/265)	Multivariable analysis • Age (per 10 years): OR = 1.53 (95% CI: 1.13 to 2.06) • Coronal deformity angular ratio (per 1 unit): OR = 1.10 (95% CI: 1.01 to 1.19) • Number of spinal levels involved (per 1 level): OR = 1.08 (95% CI: .99 to 1.17) • Lumbar-level osteotomy (yes vs no): OR = 3.30 (95% CI: 1.18 to 9.17) • Prevalence of 3CO (yes vs no): OR = 2.16 (95% CI: .77 to 6.08) • imated blood loss (per 500 cc): OR = 1.06 (95% CI: .97 to 1.15) Univariate analysis • Previous history spine surgery (yes vs no) • Preoperative neurological deficits (yes vs no) • Sagittal deformity angular ratio (per 1 unit) • Surgical approach (anterior-posterior vs posterior only)	Funding: Scoliosis research society and AOSpine international, Norton Healthcare COI: None
Zhang 2017 Retrospective cohort Study quality: Fair	Surgical indication: Congenital scoliosis (19.3%), kyphoscoliosis (74.2%), kyphosis (6.5%) Eligible: N = NR Analyzed: N = 62 Mean age (range): 16.3 (6 to 46) years Males: 45.2%	NR	• Thoracic posterior vertebral column resection	Decrease in ASIA grade at discharge. Not explicitly defined Incidence: 16.1% (10/62)	Multivariable analysis • Age (≥18 vs <18 years): OR = 8.27 (95% CI: 1.17 to 58.71) • Pulmonary function (normal vs abnormal): OR = 2.10 (95% CI: .99 to 4.48) • Pre-operative neurological status (normal vs abnormal): OR = NR, P > .05 • Blood loss (>50% vs <50%): OR = 3.05 (95% CI: 1.16 to 8.05) Univariate analysis • Cobb (main curve) (≥90 vs <90) • Operative time (≥480 vs <480 minutes) • BMI (normal vs abnormal) • Malformation type (kyphoscoliosis vs scoliosis + kyphosis) • Number of vertebrae fused (≥10 vs ≤10) • Number of vertebrae resected (≥2 vs 1) • Use of titanium mesh/cage (yes vs no) • Intraspinal deformity (yes vs no)	Funding: Science and technology innovation project in Shaanxi Province of China, natural Science Foundation of China, natural Science Basic research plan in Shaanxi Province in China, and the Youth development Project of the Army Medical Technology COI: None
Mixed surgical indications						
Chen 2012 Retrospective cohort (matched pairs)^d^ Study quality: Good	Surgical indication: Spinal degeneration (35%), spinal tumor (23%), spinal trauma (22%), spinal deformity (16%), and spinal inflammation (4%) Eligible: NR Analyzed: N = 316 Mean age: 33.33 ± 7.69 years Males: 70%	• Major and current psychiatric illnesses or cognitive deficits • Neurological function of spinal cord could not be measured correctly • Significant traumatic brain injuries • Major medical diseases • Major neurological deficits or diseases • Pregnancy • Life-threatening injuries or other concurrent injuries which prevent spinal surgery	• Decompression • Internal fixation • Bone graft • Reduction (No other information provided)	Iatrogenic SCI defined as a decrease in postoperative ASIA grade. No further details given Incidence: 25% (79/316)^e^	Multivariable analysis • Age (continuous^f^): OR = 1.08 (95% CI: 1.03 to 1.13) • Sex (male vs female): OR = 5.22 (95% CI: 1.86 to 14.62) • Hypertension (yes vs no): OR = 15.18 (95% CI: 4.50 to 51.17) • Preoperative spinal cord function (AIS A-D; better vs worse): OR = .35 (95% CI: .18 to .66) • Involved segments (more vs less): OR = 3.28 (95% CI: 1.55 to 6.92) Univariate analysis • Diabetes mellitus • Extent of compression to spinal cord on MRI (normal, decompression without ischemia and ischemia)	Funding: Ministry of Science and Technology of the People’s Republic of China COI: None
Romero-Munoz 2019 Retrospective cohort Study quality: Fair	Cases were of intra-op SCI from elective surgery, control population appear to be SCI from other presentation Surgical indication: Spinal degeneration (ie, stenosis, disc herniation, spondylolisthesis w/w/o spinal stenosis), primarily presenting with pain and radiculopathy without deficit (75%); spinal deformity (ie, scoliosis or kyphosis) (17.5%); spine fracture (3.5%); other^g^ (4%) Location of SCI Cervical (30%), thoracic (43%), and lumbar (27%) Eligible: N = 1282 Analyzed: N = 1282 Mean age (IQR): 58 (45 to 69) years Males: 54.4%	• SCI following primary or metastatic cancer surgery • SCI secondary to diagnostic procedures, epidural puncture or procedures performed during childbirth	Cervical • Discectomy and arthrodesis • Corpectomy and arthrodesis Lumbar • Laminectomy and discectomy Laminectomy and arthrodesis	Decrease in ASIA grade at discharge. Not explicitly defined Incidence: 9% (114/1282)^h^	Multivariable analysis (all adjusted for age) • Age (continuous^f^, median): OR = 1.004 (95% CI: .98 to 1.03) • Diabetes mellitus (yes vs no): OR = .70 (95% CI: .20 to 2.38) • Obesity (yes vs no): OR = .52 (95% CI: .16 to 1.69) • Hypertension (yes vs no): OR = 1.47 (95% CI: .56 to 3.86) • Dyslipidemia (yes vs no): OR = .48 (95% CI: .16 to 1.47)^i^ • Depression (yes vs no): OR = 2.69 (95% CI: .95 to 7.59) Univariate analysis • Sex (female vs male) • Spinal cord Independence measure at baseline (continuous, median) • AIS scale at baseline (absolute frequency, %)	Funding: None received COI: None
Degenerative spine disease						
Kim 2021 Retrospective cohort Study quality: Poor	Surgical indication: OPLL Eligible: N = 210 Analyzed: N = 196 Mean age: NR Males: 67.3	• Other indications for ACDF than OPLL (infection, fracture, tumor and/or inflammatory and congenital musculoskeletal disorders • Patients undergoing other concurrent surgery (eg, posterior cervical fusion, occipito-cervical fusion, atlanto-axial fusion, cranial surgery) • Inadequate medical records to confirm postoperative neurological state	• Anterior cervical discectomy with fusion with/without corpectomy	Postoperative neurological complications defined as “any new limb motor or sensory neurological deficits observed immediately post-operation”. No further details given Incidence: 7.1% (14/196)	Multivariable analysis • Sex (male vs female): OR = 1.378 (95% CI: .33 to 5.79) • Age (continuous^f^): OR = .97 (95% CI: .89 to 1.05) • BMI (continuous^f^): OR = 1.11 (95% CI: .96 to 1.29) • Compressive myelopathy prior to surgery (yes vs no): OR = 8.24 (95% CI: 1.57 to 43.38) • CCI score (categorical, 0, 1, 2, or ≥3; lower vs higher): OR = 1.02 (95% CI: .49 to 2.14) • Operation type (emergency vs elective): OR = .00 (95% CI: NR), P = .999 • Operative time (continuous^f^): OR = 1.004 (95% CI: .99 to 1.01) • Number of levels fused (1 to 2 vs ≥3): OR = 1.36 (95% CI: .59 to 3.11) • Blood loss (continuous^f^): OR = 1.00 (95% CI: .99 to 1.002) • Use of intraoperative neuromonitoring (yes vs no): OR = .14 (95% CI: .04 to .52) Univariate analysis • Race (Asian vs other)	Funding: None COI: None

3CO = 3 column osteotomy; AIS = ASIA Impairment Scale; ASD = adult spinal deformity; ASIA = American Spinal Injury Association; CCI = Charlson Comorbidity Index; COI = conflict of interest; CI = confidence interval; ICD-9 = International Classification of Diseases, Ninth Revision; IQR = interquartile range; NR = Not reported; OLF = ossification of the ligamentum flavum; OPLL = ossification of the posterior longitudinal ligament; OR = Odds Ratio; ROB = Risk of bias; SCI = Spinal cord injury.

^a^If no definition of variable is listed, the article did not provide one.

^b^Risk factors represent all variables in a given study’s univariate and multivariable analyses. Only those with effect sizes were included in multivariable analyses; all others were explicitly stated to have not been used beyond univariate analyses due to non-significance, but were otherwise assessed for an association using univariate statistical methods.

^c^Patients were selected based on the procedure performed: had to be one of the surgeries in the surgery column.

^d^Matched-pairs. Patient with SCI (n = 79) were matched with patients without SCI (n = 237) in a 1:3 ratio on the following factors: primary disease, hospital, and similar procedure.

^e^All cases, as controls were included based on lack of SCI.

^i^Text in results says dyslipidemia is only factor to show significance (OR = .34, 95% CI: .12 to .96, P = .04), however Table 9 summarizing the results of all factors in multivariable regression lists that dyslipidemia is not significant (OR = .48, 95% CI: .16 to 1.47, P = .197).

^g^Arnold Chiari, vertebroplasty, cervical epidural electrode, coccygodynia.

^f^Authors do not report details, assumed continuous.

^h^114 cases of SCI following elective surgery.

**Table 6.** Data abstraction for included studies looking at risk factors for intraoperative monitoring warnings.

Author Design	Population	Exclusion Criteria	Surgery	Intraoperative SCI definition	Risk Factors Assessed^a^ Effect Estimate (95% CI)^b^	Funding COI
Deformity						
Buckland 2018 Retrospective cohort Study quality: Poor	Surgical indication: Adolescent idiopathic scoliosis Eligible: N = 2210 Analyzed: N = 2210 Mean age: 14.7±2.1 years Males: 19.4%	NR	• Ponte osteotomy • No ponte osteotomy	Perioperative nerve root or SCI as identified by surgeon. Intraoperative neuromonitoring alerts as outcome. No further detail given Intraoperative neuromonitoring warnings defined as SSEP: Decrease ≥50% amplitude TcMEP: Decrease ≥50% amplitude Incidence: .3% (7/2210)	Multivariable analysis • Ponte osteotomy (yes vs no): OR = NR, P < .001 • Curve magnitude (continuous^c^): OR = NR, P < .001 Univariate analysis Unclear	Funding: DePuy synthes spine COI: Board membership, consultancy, royalties, grants, stocks, employment, payment for lecture, patients. No further details given

3CO = 3 column osteotomy; AIS = ASIA Impairment Scale; ASIA = American Spinal Injury Association; CCI = Charlson Comorbidity Index; COI = conflict of interest; CI = confidence interval; IQR = interquartile range; NR = Not reported; OR = Odds Ratio; ROB = Risk of bias; SCI = Spinal cord injury; SSEP = Somatosensory evoked potentials; TcMEP = Transcranial motor evoked potentials.

^a^If no definition of variable is listed, the article did not provide one.

^b^Risk factors represent all variables in a given study’s univariate and multivariable analyses. Only those with effect sizes were included in multivariable analyses; all others were explicitly stated to have not been used beyond univariate analyses due to non-significance, but were otherwise assessed for an association using univariate statistical methods.

^c^Authors do not report details, assumed continuous.

Five studies evaluated the risk of ISCI (ie, neurological decline) in the immediate postoperative period, with four studies (Fehlings 2018,^11^ Chen 2012,^19^ Romero-Munoz 2019,^21^ Zhang 2017^23^) using change in ASIA grade to assess neurological status and one study (Kim 2021)^20^ using the definition of “any new limb, motor, or sensory neurological deficit”. The sixth study (Buckland 2018)^24^ evaluated risk of IONM alerts, defined as a reduction in amplitude of 50% or more in SSEPs and/or TcMEPs to be indicative of ISCI. Results from the survey of surgeons on risk factors for ISCI was provided for context (Iyer 2022).^22^ Factors evaluated using multivariable analysis are found in Table 7. Factors assessed using univariate level are found in Table 8. Reported ISCI frequency ranged from .3% to 25% (7.1% to 25% in postoperative approaches and .3% in the study reporting on risk of IONM alert). A summary of effect estimates is presented in Table 9.

**Table 7.** Demographic, clinical, surgical, and radiographic factors potentially associated with intra-operative SCI in studies that conducted multivariable analyses.^a^

		Risk factors for Neurological Deficits					Deformity
		Deformity		Mixed Surgical Indication		Degeneration	
		Fehlings	Zhang	Chen	Romero-Muñoz	Kim	Buckland
	Intraoperative	2018	2017	2012	2019	2021	2018
	SCI definition	ASIA	ASIA	ASIA	ASIA	Unclear^b^	IONM^c^
	Surgical indication	Scoliosis	Congenital scoliosis, kyphoscoliosis, kyphosis	Spinal degeneration, tumor, trauma, deformity, inflammation	Cervical, lumbar spine injuries	OPLL	Adolescent idiopathic scoliosis
	ROB	Low	Moderate	Low	Moderate	High	High
	Risk factor						
Patient characteristics	Age	Yes	Yes	Yes	No	No	
	Sex			Yes		No	
	Hypertension			Yes	No		
	Diabetes				No		
	BMI/Obesity				No	No	
	Depression				No		
	CCI score					No	
	Dyslipidemia				No		
	Pulmonary function		Yes^d^				
Clinical	Preoperative AIS/neurological status		No	Yes			
	Combined myelopathy					Yes	
	Blood loss	No	Yes			No	
Surgical	No. of spinal levels/involved segments	No		Yes		No	
	Operation type					No	
	Operation time					No	
	Use of intra-operative monitoring					Yes	
	Lumbar-level osteotomy	Yes					
	Ponte-osteotomy						Yes
	Prevalence of 3CO	No					
Radiographic	Coronal DAR	Yes					
	Curve magnitude						Yes

3CO = 3 column osteotomy; ASIA = American Spinal Cord Injury Association; AIS = ASIA Impairment Scale; BMI = Body mass index; CCI = Charlson Comorbidity Index; DAR = deformity angular ratio; ICD-9 = International Classification of Diseases, Ninth Revision; IONM = Intra-operative neuromonitoring; OPLL = ossification of posterior longitudinal ligament; SCI = Spinal cord injury, ROB = risk of bias.

^a^“Yes” indicates that a given factor was associated with the outcome in multivariate analysis; “no” indicates that a given factor was not associated in multivariate analysis.

^b^Kim 2021 defined new neurological deficits as “any new limb motor or sensory neurological deficits observed immediately post-operation”. Every included patient was checked for their neurological status preoperatively, immediately after awaking from anesthesia, 1 day after operation, discharge period and follow up periods at outpatient clinics. No further details given.

^c^Authors report that a neuromonitoring alert was defined as a reduction in amplitude of 50% or more in SSEPs and/or tcMEPs. Increases in response latency were not included based on prior literature suggesting that it was not an independent sign of neurological injury in spine surgery.

^d^Marginal association. OR = 2.10 (95% CI 0.99 to 4.48), P = .054.

**Table 8.** All demographic, clinical, surgical, and radiographic factors explored/evaluated as prognostic factors for intraoperative SCI in univariate analyses.^a^

		Risk factors for Neurological Deficits					Risk factors for IONM^b^	Expert Consensus
		Deformity		Mixed Surgical Indication		Degeneration	Deformity	
		Fehlings	Zhang	Chen	Romero-Muñoz	Kim	Buckland	Iyer
	Intraop SCI	2018	2017	2012	2019	2021	2018	2022^c^
	definition	ASIA	ASIA	ASIA	ASIA	Unclear^d^	IONM^b^	IONM
	Surgical indication	Scoliosis	Congenital scoliosis, kyphoscoliosis, kyphosis	Spinal degeneration, tumor, trauma, deformity, inflammation	Cervical, lumbar spine injuries	OPLL	Adolescent idiopathic scoliosis	Complex spinal deformity
	ROB	Low	Moderate	Low	Moderate	High	High	NA
	Risk factor							
Patient characteristics	Age	M	M	M	M	M		√
	Sex			M	U	M		
	Race					U		
	Hypertension			M	M			
	Diabetes			U	M			
	BMI/Obesity		U		M	M		√
	Depression				M			
	Previous surgery	U						
	CCI score					M		
	SCIM				U			
	Dyslipidemia				M			
	Intraspinal deformity		U					√
	Malformation type		U					
	Non-idiopathic etiology							√
	Congenital scoliosis							√
	Congenital kyphosis							√
	Syndromic etiology							√
	Neurological comorbidity							√
	Current tethered cord							√
	Split cord malformation							√
	Presence of syrinx ≥4 mm							√
	Preoperative myelopathy							√
	Skeletal dysplasia							√
	Cardiopulmonary comorbidity							√
	Neuromuscular etiology							√
	Chronic anemia							√
	Presence of syrinx of any size							√
Clinical	Preoperative AIS/neurological status	U	M	M	U			
	Combined myelopathy					M		
	Blood loss	M	M			M		
	Pulmonary function		U					
Surgical	No. of spinal levels/involved segments	M		M		M		
	No. of levels/vertebrae fused		U					
	No. of vertebrae resected		U					
	Operation type	U				M		
	Operation time		U			M		
	Use of intraoperative monitoring					M		√
	Use of MRI			U				
	Use of titanium mesh/cage		U					
	Lumbar-level osteotomy	M						
	Ponte-osteotomy						M	
	Prevalence of 3CO	M						
	Revision surgery							√
	Prior ASF with vessel ligation							√
	Prior intradural surgery							√
	Absence of baseline IONM data, or poor-quality data							√
	Revision surgery							√
	Prior ASF with vessel ligation							√
	Prior intradural surgery							√
	Absence of baseline IONM data, or poor-quality data							√
	Hematocrit <28							√
	Higher ASA class							√
	Lack of preoperative HGT in an appropriate patient							√
	Prior fusion							√
	Intraoperative adjustment/movement of patient							√
Radiographic	Coronal DAR	M						√
	Sagittal DAR	U						√
	Cobb curve		U					
	Curve magnitude						M	√
	Total DAR							√
	Scoliosis with hyperkyphosis							√
	Bayoneted spine							√
	High rate of deformity progression							√
	Type 2 spinal cord shape							√
	Type 3 spinal cord shape							√
	Structural curve							√

3CO = 3 column osteotomy; ASIA = American Spinal Cord Injury Association; AIS = ASIA Impairment Scale; BMI = Body mass index; CCI = Charlson Comorbidity Index; DAR = deformity angular ratio; ICD-9 = International Classification of Diseases, Ninth Revision; OPLL = ossification of posterior longitudinal ligament; SCI = Spinal cord injury; SCIM = Spinal Cord Independence Measure.

^a^U represents factors explored only in univariate analyses; M represents factors explored at the multivariable level. Checkmarks indicate factors considered amongst a consensus survey.

^d^Kim 2021 defines new neurological deficits as “any new limb motor or sensory neurological deficits observed immediately post-operation”. No further details given.

^b^Authors report that a neuromonitoring alert was defined as a reduction in amplitude of 50% or more in SSEPs and/or tcMEPs, but that increases in response latency were not included based on prior literature suggesting that it was not an independent sign of neurological injury in spine surgery.

^c^Article included for context. Reports survey consensus amongst 15 experts and is not a patient-based multivariable assessment of factors associated with SCI. Checkmarks indicate all risk factors considered.

**Table 9.** Summary of Effect Estimates for Risk Factors for Intra-operative SCI in Studies Using Multivariable Analysis.

Prognostic Factor	Number of Studies (Number of Patients)Factor Details: OR (95% CI)
Demographic	
Age	**Deformity** Fehlings, 2018 (N = 272) Age (per 10 years): OR = 1.53 (95% CI 1.13 to 2.06), P = .05 Mean age: 56.8 ± 15.4 years Zhang, 2017 (N = 62) Age (≥18 vs <18 years): OR = 8.27 (95% CI 1.17 to 58.71), P = .035 Mean age: 16.3 ± 6.4 years **Mixed** Chen, 2012 (N = 316) Age (continuous^a^): OR = 1.08 (95% CI 1.03 to 1.13), P < .001 Mean age: 43.15 ± 6.47 years Romero-Muñoz, 2019 (N = 1282) Age (continuous^a^): OR = 1.004 (95% CI 0.98 to 1.03), P = .759 Median age (IQR): 58 (45 to 69) years **Degeneration** Kim, 2021 (N = 210) Age (continuous^a^): OR = .97 (95% CI 0.89 to 1.05), P = .446 Mean age: 57 ± 12.2 vs 58 ± 12 years^d^
Sex	**Mixed** Chen, 2012 (N = 316) Sex (male vs female): OR = 5.22 (95% CI 1.86 to 14.62), P = .002 **Degeneration** Kim, 2021 (N = 196) Sex (male vs female): OR = 1.378 (95% CI 0.33 to 5.79), P = .661
Hypertension	**Mixed** Chen, 2012 (N = 316) Hypertension (yes vs no): OR = 15.18 (95% CI 4.50 to 51.17), P < .001 Romero-Muñoz, 2019 (N = 1282) Hypertension (yes vs no): OR = 1.47 (95% CI 0.56 to 3.86), P = .436
Diabetes	**Mixed** Romero-Muñoz, 2019 (N = 1282) Diabetes mellitus (yes vs no): OR = .70 (95% CI 0.20 to 2.38), P = .562
BMI/Obesity	**Mixed** Romero-Muñoz, 2019 (N = 1282) Obesity (yes vs no): OR = .52 (95% CI 0.16 to 1.69), P = .276 **Degeneration** Kim, 2021 (n = 196) BMI (continuous^a^): OR = 1.11 (95% CI 0.96 to 1.29), P = .154
Depression	**Mixed** Romero-Muñoz, (N = 1282) Depression (yes vs no): OR = 2.69 (95% CI 0.95 to 7.59), P = .061
CCI score	**Degeneration** Kim, 2021 (n = 196) CCI score (categorical, 0, 1, 2, or ≥3; lower vs higher): OR = 1.02 (95% CI 0.49 to 2.14), P = .953
Dyslipidemia	**Mixed** Romero-Muñoz, (N = 1282) Dyslipidemia (yes vs no): OR = .48 (95% CI 0.16 to 1.47), P = .197
Pulmonary function	**Deformity** Zhang, 2017 (N = 62) Pulmonary function (abnormal vs normal): OR = 2.10 (95% CI 0.99 to 4.48), P = .054
Clinical	
Preoperative AIS/Neurological status	**Deformity** Zhang, 2017 (N = 62) Pre-operative neurological status (normal vs abnormal): OR = NR, P > .05 **Mixed** Chen, 2012 (N = 316) Pre-operative AIS status (A-D; better vs worse): OR = .35 (95% CI 0.18 to .66), P = .001
Combined myelopathy	**Degeneration** Kim, 2021 (N = 196) Combined myelopathy prior to surgery (yes vs no): OR = 8.24 (95% CI 1.57 to 43.38), P = .013
Blood loss	**Deformity** Fehlings, 2018 (N = 265) Estimated blood loss (per 500 cc): OR = 1.06 (95% CI 0.97 to 1.15), P = .179 Zhang, 2017 (N = 62) Estimated blood loss^b^ (>50% vs <50%): OR = 3.05 (95% CI 1.16 to 8.05), P = .024 **Degeneration** Kim, 2021 (N = 196) Estimated blood loss (continuous^a^): OR = 1.00 (95% CI 0.99 to 1.002), P = .862
Surgical	
Number of spinal levels	**Deformity** Fehlings, 2018 (N = 265) Number of spinal levels involved (per 1 level): OR = 1.08 (95% CI 0.99 to 1.17), P = .091 **Degeneration** Kim, 2021 (N = 196) Number of levels fused (1 to 2 vs ≥3): OR = 1.36 (95% CI 0.59 to 3.11), P = .470 **Mixed** Chen, 2012 (N = 316) Number of involved segments (more vs less): OR = 3.28 (95% CI 1.55 to 6.92), P = .002
Operation type	**Degeneration** Kim, 2021 (N = 196) Operation type (emergency vs elective): OR = .00 (95% CI NR), P = .999
Operation time	**Degeneration** Kim, 2021 (N = 196) Operation time (continuous^a^): OR = 1.004 (95% CI 0.99 to 1.01), P = .238
Use of intra-operative monitoring	**Degeneration** Kim, 2021 (N = 196) Use of intra-operative neuromonitoring (yes vs no): OR = .14 (95% CI 0.04 to .52), P = .003
Lumbar-level osteotomy	**Deformity** Fehlings, 2018 (N = 265) Lumbar-level osteotomy (yes vs no): OR = 3.30 (95% CI 1.18 to 9.17), P = .022
Ponte-osteotomy	**Deformity**^a^ Buckland, 2018 (N = 2210) Ponte osteotomy (yes vs no): OR = NR, P < .001
Prevalence of 3 column osteotomy	**Deformity** Fehlings, 2018 (N = 265) Prevalence of 3CO (yes vs no): OR = 2.16 (95% CI 0.77 to 6.08), P = .143
Radiographic	
Coronal deformity angular ratio	**Deformity** Fehlings, 2018 (N = 265) Coronal deformity angular ratio (per 1 unit): OR = 1.10 (95% CI 1.01 to 1.19), P = .037
Curve magnitude	**Deformity**^a^ Buckland, 2018 (N = 2210) Curve magnitude (continuous^a^) OR = NR, P < .001

3CO = 3 Column Osteotomy; CCI = Charlson Comorbidity Index; IQR = Interquartile range; OR = odds ratio; CI = confidence interval; AIS = ASIA Impairment Scale; BMI = body mass index; NR = not reported.

^a^Authors do not report details, variable is assumed to be continuous.

^b^EBL, the ratio between circulating and lost blood.

^c^Risk factors for intra-operative neuromonitoring defined as a reduction in amplitude of 50% or more in SSEPs and/or tcMEPs. Increases in response latency were not included based on prior literature suggesting that it was not an independent sign of neurological injury in spine surgery.

^d^Represents patients that received intra-operative monitoring group vs patients that did not receive intra-operative monitoring.

Three patient population groups were identified in the included studies: those with deformity, those with various indications for spine surgery, and those with degenerative disease.

Evidence for the risk factors for neurological deficits in patients with deformities was derived from one good-quality, prospective cohort (Fehlings 2018, N = 265)^11^ on scoliosis in adults, and one fair-quality, retrospective cohort (Zhang 2017, N = 62)^23^ in patients with congenital scoliosis (19%), kyphoscoliosis (74%), and kyphosis (7%). Another poor-quality retrospective cohort (N = 2210) (Buckland 2018)^24^ described risk factors for IONM alerts in adolescent patients with idiopathic scoliosis.

Studies in patients with surgery for deformity found an increased risk of ISCI with older age, higher blood loss, surgical technique related factors like the requirement for osteotomy, and radiographic factors including coronal deformity angular ratio (DAR) and curve magnitude. For patients with “mixed” indications, one good-quality, retrospective cohort (N = 316) (Chen 2012)^19^ reported on patients with spinal degeneration (35%), tumor (23%), trauma (22%), deformity (16%), and inflammation (4%), while one fair-quality retrospective cohort (N = 1282) (Romero-Munoz 2019)^21^ reported on patients with spinal degeneration (75%), deformity (18%), fractures (4%), or other rare injuries (4%). In this latter study, authors did not describe the group to which patients with ISCI were compared to. It appears that patients receiving elective surgery who experienced ISCI were compared to those with other causes of SCI. Studies enrolling patients undergoing spine surgery for a variety of indications found that older age, male sex, hypertension, depression, and a higher number of operative spinal levels were associated with ISCI.

Overall, two studies (Fehlings,^11^ Chen^19^) were rated good, two (Zhang 2017,^23^ Romero-Munoz 2020^21^) were rated fair, and two (Kim 2021,^20^ Buckland 2019^24^) were rated poor quality One poor quality retrospective cohort (Kim 2021)^20^ reported on patients with degeneration and focused solely on patients with ossification of posterior longitudinal ligament (OPLL) (Table 10). Common methodological concerns included retrospective collection of complications (five of the six studies were retrospective study designs) and unclear or unknown study attrition. Other, less frequent concerns included inadequate description of inclusion/exclusion criteria and unclear validity and/or reliability of the measurement methods for prognostic factors and/or confounders. For the studies where the indication for surgery was degeneration, male sex, obesity, Charleston Comorbidity Index (CCI), combined myelopathy, a higher number of operative spinal levels, and increased time of surgery were associated with ISCI.

**Table 10.** Strength of evidence (SOE) table assessing risk factors for intra-operative SCI in prognostic studies.

Prognostic factors	No. Studies (no. patients)	Overall Quality	Conclusions^a^	Study Limitations	Serious Inconsistency	Serious Indirectness	Serious Imprecision	Publication Bias
Demographic								
Age	**Deformity** 2 (N = 327) Fehlings 2018 Zhang 2017	MODERATE	**Deformity** Increasing age was associated with increased odds of experiencing ISCI	Low	Unknown^†^	No	No	Unknown
	**Mixed** 2 (N = 1598) Chen 2012 Romero-Muñoz 2019	LOW	**Mixed** Increasing age was associated with increased odds in one study; no association in a second, larger study	Moderate	No	No	No	Unknown
	**Degeneration** 1 (N = 196) Kim 2021	VERY LOW	**Degeneration** Age was not associated with increased odds of experiencing an ISCI	High	Unknown	No	No	Unknown
Sex	**Mixed** 1 (N = 316) Chen 2012	LOW	**Mixed** Males had increased odds of experiencing an ISCI	Low	Unknown	No	Yes	Unknown
	**Degeneration** 1 (N = 196) Kim 2021	VERY LOW	**Degeneration** Sex was not associated with increased odds of experiencing an ISCI	High	Unknown	No	Yes	Unknown
Hypertension	**Mixed** 2 (N = 1598) Chen 2012 Romero-Muñoz 2019	LOW	**Mixed** Hypertension was not associated with increased odds of experiencing an ISCI in a larger study; hypertension was associated with a large increase in odds in a second study, but confidence intervals were extremely wide	Moderate	Yes	No	Yes	Unknown
Diabetes	**Mixed** 1 (N = 1282) Romero-Muñoz 2019	LOW	**Mixed** Diabetes was not associated with increased odds of experiencing an ISCI	Moderate	Unknown	No	No	Unknown
BMI/Obesity	**Mixed (obesity)** 1 (N = 1282) Romero-Muñoz 2019	LOW	**Mixed** BMI/obesity was not associated with increased odds of experiencing an ISCI	Moderate	Unknown	No	Yes	Unknown
	**Degeneration (BMI)** 1 (N = 196) Kim 2021	VERY LOW	**Degeneration** BMI/obesity was not associated with increased odds of experiencing an ISCI	Yes	Unknown	No	No	Unknown
Depression	**Mixed** 1 (N = 1282) Romero-Muñoz 2019	LOW	**Mixed** Depression was not associated with increased odds of experiencing a ISCI	Moderate	Unknown	No	Yes	Unknown
CCI score	**Degeneration** 1 (N = 196) Kim 2021	VERY LOW	**Degeneration** CCI scores at baseline were not associated with increased odds of experiencing an ISCI	High	Unknown	No	No	Unknown
Dyslipidemia	**Mixed** 1 (N = 1282) Romero-Muñoz 2019	LOW	**Mixed** Dyslipidemia was not associated with increased odds of experiencing an ISCI	Moderate	Unknown	No	No	Unknown
Pulmonary function	**Deformity** 1 (N = 62) Zhang 2017	VERY LOW	**Deformity** Abnormal pulmonary function was associated with increased odds of experiencing an ISCI	Yes	Unknown	No	Yes	Unknown
**Clinical**								
Preoperative AIS/Neurological status	**Deformity** 1 (N = 62) Zhang 2017	VERY LOW	**Deformity** Preoperative neurological status (normal vs abnormal) was not associated with increased odds of experiencing an ISCI	Moderate	Unknown	No	Yes (CI not provided)	Unknown
	**Mixed**^c^ 1 (N = 316) Chen 2012	MODERATE	**Mixed**^c^ Better preoperative AIS was associated with decreased odds of experiencing an ISCI	Low	Unknown	No	No	Unknown
Combined myelopathy	**Degeneration** 1 (N = 196) Kim 2021	VERY LOW	**Degeneration** Presence of compressive myelopathy prior to surgery was associated with a large, increased odds of experiencing an ISCI; confidence interval was extremely wide	High	Unknown	No	Yes	Unknown
Estimated blood loss	**Deformity** 2 studies (N = 327) Fehlings 2018 Zhang 2017	MODERATE	**Deformity** EBL (per 500 cc) was not associated with increased odds of experiencing an ISCI in the larger, good-quality study; the fair-quality study found EBL ≥50% vs <50% (ie, ratio between circulating and lost blood) was associated with increased odds of experiencing an ISCI, but confidence interval was wide	Low	Unknown^§^	No	No	Unknown
	**Degeneration** 1 (N = 196) Kim 2021	VERY LOW	**Degeneration** EBL (continuous) was not associated with increased odds of experiencing an ISCI	High	Unknown	No	No	Unknown
**Surgical**								
Number of spinal levels	**Deformity** 1 (N = 265) Fehlings 2018	MODERATE	**Deformity** Number of spinal levels involved (1 per level) was not associated with increased odds of experiencing an ISCI	Low	Unknown	No	No	Unknown
	**Degeneration** 1 (N = 196) Kim 2021	VERY LOW	**Degeneration** Number of levels fused (continuous) was not associated with increased odds of experiencing an ISCI	High	Unknown	No	Yes	Unknown
	**Mixed** 1 (N = 316) Chen 2012	LOW	**Mixed** Increasing number of involved segments was associated with a large increased odds of experiencing an ISCI, but the confidence interval was wide	Low	Unknown	No	Yes	Unknown
Lumbar-level osteotomy	**Deformity** 1 (N = 265) Fehlings 2018	LOW	**Deformity** Lumbar-level osteotomy was associated with large increased odds of experiencing an ISCI, but the confidence interval was wide	Low	Unknown	No	Yes	Unknown
Ponte-osteotomy	**Deformity** 1 (N = 2210) Buckland 2018	VERY LOW	**Deformity** Ponte-osteotomy was associated with increased odds of an IONM alert	High	Unknown	No	Yes (OR/CI not provided)	Unknown
Prevalence of 3CO	**Deformity** 1 (N = 265) Fehlings 2018	LOW	**Deformity** 3-Column osteotomy was not associated with increased odds of experiencing an ISCI; the confidence interval was wide	Low	Unknown	No	Yes	Unknown
Operation type	**Degeneration** 1 (N = 196) Kim 2021	VERY LOW	**Degeneration** Operation type (emergency vs elective) was not associated with increased odds of experiencing an ISCI	High	Unknown	No	Yes (CI not provided)	Unknown
Operation time	**Degeneration** 1 (N = 196) Kim 2021	VERY LOW	**Degeneration** Operation time (continuous) was not associated with increased odds of experiencing an ISCI	High	Unknown	No	No	Unknown
Use of intra-operative monitoring	**Degeneration** 1 (N = 196) Kim 2021	VERY LOW	**Degeneration** Use of intra-operative monitoring was associated with a large decrease in odds of experiencing an ISCI	High	Unknown	No	No	Unknown
**Radiographic**								
Coronal DAR	**Deformity** 1 (N = 265) Fehlings 2018	MODERATE	**Deformity** Coronal DAR (per 1 unit) was associated with increased odds of experiencing an ISCI	Low	Unknown	No	No	Unknown
Curve magnitude	**Deformity** 1 (N = 2210) Buckland 2018	VERY LOW	**Deformity** Curve magnitude (coronal and sagittal Cobb) was associated with increased odds of IONM alerts	High	Unknown	No	Yes (OR/CI not provided)	Unknown

3CO = 3 Column Osteotomy; CCI = Charlson Comorbidity Index; CI = confidence interval; DAR = Deformity Angular Ratio; OR = odds ratio.

^a^Conclusions addressed association between prognostic factor and increased risk of intra-operative/immediate postoperative neurological deficit or decline (measured via ASIA grade) in all studies except for Buckland 2018 which evaluated the association is between the factor and the risk of intra-operative monitoring alerts.

^b^Age modeled differently: Fehlings et al evaluated age per 10-year increments and Zhang et al evaluated age dichotomized into ≥18 vs <18 years. Also, the mean age of the study populations differed: 56.8 years (SD 15.4, range 18-81) for Fehlings et al and 16.3 years (SD 6.4, range 6-46 years) for Zhang et al.

^c^Large proportion (almost 50%) of patients had AIS grade B.

^d^Different ways of measuring estimated blood loss in the two studies: per 500 cc (Fehlings et al) and as the ratio between circulating and lost blood dichotomized into ≥50% vs <50% (Zhang et al). Populations also appear to be different.

## Patient Specific Risk Factors for ISCI

Five studies reported on age as a risk factor for ISCI (Fehlings 2018,^11^ Romero Munoz 2019,^21^ Zhang 2017,^23^ Chen 2012,^19^ Kim 2021^20^). Four studies reported increased risk of ISCI with increased age (Fehlings 2018,^11^ Romero Munoz 2019,^21^ Zhang 2018,^23^ Chen 2012^19^). Two of these studies reported this as a significant association (Zhang 2017,^23^ Chen 2012^19^), while in the other two studies the association was not significant (Fehlings 2018,^11^ Romero Munoz 2019^21^). In one study, a slightly decreased risk of ISCI was associated with increased age (OR = .97), which was not significant (P > .05) (Kim 2021^20^). Male sex was associated with increased risk of ISCI in two studies. The association was significant in one study, while not significant in the other (Chen 2012,^19^ Kim^20^). Hypertension was associated with a significantly increased risk of ISCI in one study, and a non-significantly increased risk of ISCI in another study (Chen 2012,^19^ Romero Munoz 2019^21^). Diabetes Mellitus was associated with a non-significant decreased risk of ISCI in one study (Romero Munoz 2019^21^). Obesity was associated with an increased but non-significant risk of ISCI in one study (Romero Munoz 2019^21^). Another study reported a non-significant increased risk of ISCI with increasing body mass index (BMI) (Kim^20^). Clinical depression was associated with a non-significant increased risk of ISCI (Romero Munoz 2019^21^). CCI was also associated with a non-significant increased risk of ISCI (Kim^20^). Dyslipidemia has a non-significant association with decreased risk of ISCI in one study (Romero Munoz 2019^21^). Worse pulmonary function was reported to have increased risk of ISCI in one study that was non-significant (Zhang 2017^23^).

### Clinical Risk Factors for ISCI

Pre-operative neurological status was reported as a risk factor in two studies (Chen 2012,^19^ Zhang 2017^23^). In one study, a better pre-operative neurological status was associated with a significantly decreased risk of ISCI (Chen 2012^19^). In a second study, the association was not significant, although it was associated with a decreased risk (Zhang 2017^23^). Combined myelopathy was associated with a significantly increased risk of ISCI in one study (Kim 2021^20^).

Blood loss was associated with an increased risk of ISCI in two studies (Fehlings 2018,^11^ Zhang 2017^23^). The association was significant in one study, and non-significant in the other (Zhang 2017,^23^ Fehlings 2018^11^). In a third study, blood loss had equivocal (OR = 1) association with ISCI that was not significant (Kim 2021^20^).

### Surgical Risk Factors for ISCI

A higher number of spinal levels was associated with increased risk of ISCI in two studies and with a decreased risk in one study. The increased risk was significant in one study and non-significant in the second study. The decreased risk was non-significant (Fehlings 2018,^11^ Kim 2021,^20^ Chen 2012^19^). Increasing operation time was associated with increased risk of ISCI, however this was not significant (Kim 2021^20^). Lumbar level osteotomy was associated with a significantly increased risk of ISCI. In the same study, prevalence of three level osteotomy had a non-significant increased risk of ISCI (Fehlings 2018^11^). In another study, ponte-osteotomy was associated with a significant increased risk of ISCI (Buckland 2018^24^). Use of IONM was associated with a significant decreased risk of ISCI in one study (Kim 2021^20^)

### Radiological Risk Factors ISCI

One study (Fehlings 2018)^11^ on patients with scoliosis reported a greater odds of postoperative neurological deficit per 1 unit increase of coronal deformity angular ratio (DAR). One study (Buckland 2018)^24^ in patients with adolescent scoliosis found a significant positive association between spinal curve magnitude and IONM alerts but did not report an effect estimate.

In addition to risk factors identified in the systematic review, the GDG proposed seven characteristics of high-risk patients for ISCI. These included rigid thoracic curve with high deformity angular ratio, revision congenital deformity with significant cord compression and myelopathy, extrinsic intradural or extradural lesion with cord compression and myelopathy, intramedullary tumor, unstable fractures (bilateral facet dislocation and disc herniation, extension distraction injury with ankylosing spondylitis, ossification of posterior longitudinal ligament (OPLL) with severe cord compression, and moderate to severe myelopathy. These risk factors were thoroughly discussed and voted upon by the GDG. Eventually, these risk factors were accepted as high risk after a unanimous vote according to the Delphi Process.

### Quality (Strength) of Evidence

The overall quality (strength) of evidence for risk factors for ISCI based on multivariate analyses was low or very low for most factors across surgical conditions (Table 10). Increased odds for ISCI varied by surgical indication/population (eg, deformity). In patients undergoing surgery for spinal deformity, there was moderate evidence of increased odds for ISCI in patients with increasing age and increasing coronal DAR. There was moderate evidence that estimated blood loss and the number of spinal levels were not associated with increased odds of ISCI in the same population. There was moderate evidence that better preoperative AIS was associated with decreased odds of ISCI in a mixed population.

### Consensus Summary of Risk Factors for Intraoperative Spinal Cord Injury

Based on the knowledge synthesis summarized above and a consensus-based Delphi approach with the GDG, a proposed list of risk factors for ISCIS was defined (Table 11).

**Table 11.** Factors which increase the risk for intra-operative spinal cord injury.

High risk deformity: rigid thoracic curve with high deformity Angular Ratio (DAR)
Revision congenital deformity with significant cord compression & myelopathy
Extrinsic lesion with cord compression & myelopathy
Intramedullary tumor
Unstable fractures: B/L facet dislocation and disc herniation
Extension distraction injury with ankylosing spondylitis
OPLL with severe cord compression and moderate to severe myelopathy

## Discussion

The study of ISCI has been limited to date. Consequently, prior to this Focus issue, a paucity of evidence exists to guide clinicians in the decision-making surrounding patients who sustain an ISCI. This article has sought to identify the definition, frequency, risk factors, and management of ISCI through a scoping and systematic review of the existing literature.

### Definition of ISCI

To define ISCI a scoping review was conducted in which studies were reviewed and divided into two groups based on whether they focused on deformity or tumor surgery. Three studies by Lenke et al,^12^ Fehlings et al^11^ and Kato et al^10^ were centered around outcomes after adult deformity surgery. These outcomes were reported with respect to LEMS, where a major decline was defined as a loss of more than 5 points, as this correlated to a deficit in 3 or more myotomes for 90% of the major decline group.^10^ The aforementioned deficits were assessed at various time points postoperatively and therefore represent outcomes as a result of surgery rather than the natural history of the disease. The ASIA assessment is based on scoring of each key myotome from 0-5 on the MRC scale. The LEMS is the sum of all myotomes in the lower extremities bilaterally – this has been shown to correlate with ambulatory ability.^11^

Within tumor-based surgery, six studies provided definitions of ISCI. Each of these studies aimed to demonstrate the role of IONM in spinal cord tumor resection surgery. The definition of ISCI varied from a decrease in McCormick grade, MRC grade or neuromonitoring signal changes, with periods of assessment ranging from immediately postoperatively to outpatient follow up. The McCormick grade defines a patient’s neurological impairment based on motor and sensory symptoms and functional status. This was used by Harel,^13^ Korn^15^ and Sala^17^ to define ISCI resulting from spinal tumor resection surgery. Overall, the definition “a new or worsening neurological deficit attributable to spinal cord dysfunction during spine surgery that is diagnosed intra-operatively via neurophysiologic monitoring or immediately post-operatively based on clinical assessment” settled on for the purposes of this review is based on a combination of the studies included in this review and GDG recommendations. This definition encompasses both the role of IONM and the clinical impact on the patient. The true impact of an ISCI is dependent on how the SCI affects the patient clinically; therefore, while the definition of ISCI relates to changes in neuromonitoring, further investigation is required to determine how IONM signals translate to clinical findings. Additionally, the review identified the most commonly used threshold for IONM indicative of ISCI as loss of 50% or more signal on IONM. The above-mentioned definition was reviewed by the GDG in the context of evidence collected from the scoping review. Changes were proposed and voted upon, in accordance with the Delphi Process. The use of wake-up test to detect ISCI was added to the definition as it was proposed that IONM is not readily available across the globe. This change was upheld after the voting process.

Based on the knowledge synthesis and a Delphi-based approach with the GDG, the following definition of Intraoperative Spinal Cord Injury was proposed:

“a new or worsening neurological deficit attributable to spinal cord dysfunction during spine surgery that is diagnosed intra-operatively via neurophysiologic monitoring or via wake-up test, or immediately post-operatively based on clinical assessment”.

### Frequency of ISCI

The frequency of ISCI varied across pathology and spinal level, as seen in Table 3. The data from this review suggested that the frequency of ISCI may be greater in tumor surgery with a range of 0-61% and persistent deficits up to 27%.^16^ Of the 13 studies reporting on tumor surgery and IONM, one reported exclusively on extradural tumors (frequency of 1.97% with post-operative deficits), three on intradural extramedullary tumors (up to 16.5% with post-operative deficits), five on intramedullary tumors (up to 71.4% with ISCI) and the remainder on tumors in varied locations. This higher frequency of ISCI could be due in part to pre-existing deficits resulting from tumors within or abutting the cord.^23,25,26^

### Risk Factors of ISCI

The most commonly reported risk factors identified in this systematic review included older age, male sex, hypertension, pre-operative neurological status, blood loss, higher BMI, and number of spinal levels operated on. Older age, male sex, hypertension, combined myelopathy, blood loss, ponte-osteotomy, DAR, and curve magnitude were identified as factors with a statistically significant increased risk of ISCI. A better pre-operative neurological status and use of IONM were identified as factors associated with a significantly decreased risk of ISCI.

One of the most commonly cited risk factors was a decreased preoperative neurological status that can be considered evidence of preoperative spinal cord dysfunction.^21^ This can be attributed to the fact that a damaged cord is considered more vulnerable (or potentially “with less reserve”) to further insult.^19^ Furthermore, it is possible that tumors result in intrinsic cord damage and that patients undergoing surgical resection may have more deficits preoperatively. These preoperative deficits may therefore increase the risk of ISCI as the spinal cord may be more susceptible to ischemia (on top of the obvious fact that the surgical approach often requires dissection through parts of the spinal cord that may be unaffected preoperatively).

The frequency of deficits differed by spinal level, with the lumbar spine representing the greatest frequency of ISCI. Some literature has suggested that the sparse blood supply in the thoracolumbar region may contribute to the relatively increased risk of ischemia and subsequent increased frequency of ISCI in this region.^26^ Importantly, while the lumbar region has the highest reported frequency of ISCI, studies have identified a potential for injury to the cord during surgery at all spinal levels. Additionally, while injury at a lumbar spinal level may not, in the strictest definition, mean injury to the lumbar spinal cord but rather to the cauda equina (ie, nerve root injury), for the purpose of this review, it was considered as ISCI as the etiology of the injury was still iatrogenic during spine surgery.

Several mechanisms have been suggested to describe the etiology of ISCI. Studies have proposed that injury is the result of direct mechanical trauma to the cord or from spinal cord ischemia.^27,28^ Mechanical trauma can result from the placement of instrumentation or compression from surrounding structures such as the ligamentum flavum or the intervertebral disc. Vitale et al also discussed the implications of ischemia on ISCI and demonstrated that those with cardiopulmonary comorbidities were more likely to sustain ISCI as detected by IONM. This finding was not reported by the studies assessed in our scoping review – however, blood loss was described as a risk factor for ISCI in two studies and may potentially be a surrogate for cord perfusion.^28^ Additionally, Zhang et al showed that pulmonary function had an increased risk of ISCI (although not significant).^23^

Older age was also reported as a risk factor for ISCI, presumably due to both the increased likelihood of postoperative complications and because of decreased neural tissue resilience with age.^19^ Depression and CCI were associated with an increased, but non-significant, risk of ISCI. Hypertension showed a positive association with ISCI in one study but not another. As such, while this scoping review has identified several risk factors of ISCI, there was not enough evidence to establish the association between patients, surgical and disease characteristics, and risk of ISCI. Further, it is not feasible to interpret age and co-morbidities in isolation as risk factors of ISCI, as these factors are related to each other. In future studies, authors could evaluate the association of frailty and risk of ISCI, as frailty index includes age as well as co-morbidities and can serve as a more comprehensive parameter.

Importantly, Kim et al reported a significant negative association between ISCI and the use of IONM (OR .14, P = .003).^20^ Specifically, this finding indicates that when IONM was used during surgery, the risk of ISCI was significantly decreased. Use of IONM is a modifiable factor unlike many other factors such as age and co-morbidities. IONM can detect ISCI in real time during surgery, which can notify the surgical team to take actions to minimize or even reverse the deficit when still possible.^23^

The curve magnitude and DAR ratios were identified as high-risk factors for ISCI. More significant deformity can result in kinking or stretching of the cord and alter its hemodynamic supply.^23^ These surgeries are naturally associated with a higher risk of ISCI due to direct manipulation of the neural elements, acute change in spinal canal alignment, the use of extensive spinal instrumentation and vascular insufficiency from stretch of the anterior spinal artery or over-shortening of the spinal column.^1,2,13^ Male sex was also shown to be a risk factor by three of the included studies. Chen et al attributed this to a potential protective effect of estrogen and progesterone in female patients.^19^ Not all studies, however, reported a statistically significant association between sex and ISCI.

There are several limitations to this knowledge synthesis. Firstly, the majority of the studies available on this topic were rated as low quality. As there was previously no single definition, method, or criteria to define ISCI, there is variation in the reported frequency and risk factors of ISCI. Further, it is possible that not all included studies documented neurological score preoperatively using standard grading methods. There are also several strengths to our review. It is the first comprehensive and systematic knowledge synthesis that evaluates the frequency and risk factors of ISCI and provides a uniform definition. The methods used to conduct the knowledge synthesis were rigorous and abided by current standards. Additionally, the quality of the studies included was ascertained and reported. Specifically, the definition was thoroughly reviewed, debated, and voted upon by the GDG after the scoping review, and was formed after a unanimous vote, following the Delphi Process.

In summary, a comprehensive definition of ISCI was provided using the evidence gathered in this knowledge synthesis and input from GDG. This standardization of nomenclature for ISCI will enable future studies to better quantify the incidence of major neurological deficits, identify relevant risk factors and assess treatment protocols for ISCI management. Furthermore, by combining the results on risk factors with the frequency data, and recommendations by GDG, this review identified a subset of patients at “higher risk” for ISCI. These patients include older patients, those with high grade tumors causing compression, severe rigid deformity requiring multiple osteotomies, or structural pathologies causing myelopathy (eg, OPLL) and those undergoing revision surgery.

While, in theory, the risk of ISCI can never be eliminated, there can be strategies developed to mitigate and minimize it. This further raises the question of the role of IONM, management strategies in the event of loss on IONM, and a potential care pathway to mitigate the adverse event.

## Conclusion

After a comprehensive mixed-methods knowledge synthesis and expert opinion input using the Delphi Method, a consensus-based definition for ISCI was developed. It has been identified that most studies have reported that a decrease of 50% or more on IONM parameter can be considered indicative of ISCI. Additionally, several clinical, surgical, and radiological risk factors for ISCI have been identified. The results synthesized in this manuscript will supplement clinicians’ knowledge of the frequency and risk factors for ISCI in order to inform decision making regarding prevention and management strategies. A uniform, consensus-based definition of ISCI will also aid in optimizing future research on this topic. Additionally, this review further raises the question of the role of IONM, management strategies in the event of an IONM alert, and a potential care pathway to manage ISCI.

## Supplemental Material

Supplemental Material - Definition, Frequency and Risk Factors for Intra-Operative Spinal Cord Injury: A Knowledge Synthesis Supplemental Material for Definition, Frequency and Risk Factors for Intra-Operative Spinal Cord Injury: A Knowledge Synthesis by Michael G. Fehlings, Ayesha Quddusi, Andrea C. Skelly, Erika D. Brodt, Ali Moghaddamjou, Anahita Malvea, Nader Hejrati, Nisaharan Srikandarajah, Mohammed Ali Alvi, Shay Stabler-Morris, Joseph R. Dettori, Lindsay A. Tetreault, Nathan Evaniew and Brian K. Kwon in Global Spine Journal
