# Supplementary material for: Definition, Frequency and Risk Factors for Intra-Operative Spinal Cord Injury: A Knowledge Synthesis
Source: Global Spine J. 2024 Mar 25;14(3 Suppl):80S–104S. doi: 10.1177/21925682231190613 (PMC10964886; doi:10.1177/21925682231190613)
Supplement: Supplemental Material - Definition, Frequency and Risk Factors for Intra-Operative Spinal Cord Injury: A Knowledge Synthesis [file sj-pdf-1-gsj-10.1177_21925682231190613.pdf]

Appendix A. Excluded studies.

List of Select Excluded Studies and Rationale

	Citation	Reason for exclusion
1	*Ahn H, Fehlings MG. Prevention, identification, and treatment of perioperative spinal cord injury. Neurosurg Focus. 2008;25(5):E15. doi: 10.3171/FOC.2008.25.11.E15. PMID: 18980475.*	*Does not use multivariable regression to assess risk factors*
2	*Alosh H, Parker SL, McGirt MJ, Gokaslan ZL, Witham TF, Bydon A, Wolinsky JP, Sciubba DM. Preoperative radiographic factors and surgeon experience are associated with cortical breach of C2 pedicle screws. J Spinal Disord Tech. 2010 Feb;23(1):9-14. doi: 10.1097/BSD.0b013e318194e746. PMID: 20068474.*	*Does not use multivariable regression to assess risk factors*
3	*Bejjani GK, Nora PC, Vera PL, Broemling L, Sekhar LN. The predictive value of intraoperative somatosensory evoked potential monitoring: review of 244 procedures. Neurosurgery. 1998 Sep;43(3):491-8; discussion 498-500. doi: 10.1097/00006123-199809000-00050. PMID: 9733304.*	*Ineligible study design for Key Question, e.g., case series, modeling (e.g., prediction models, thresholds/ROC, etc.)*
4	*Bridwell KH, Lenke LG, Baldus C, Blanke K. Major intraoperative neurologic deficits in pediatric and adult spinal deformity patients. Incidence and etiology at one institution. Spine (Phila Pa 1976). 1998 Feb 1;23(3):324-31. doi: 10.1097/00007632-199802010-00008. PMID: 9507620.*	*Does not use multivariable regression to assess risk factors*
5	*Chen J, Shao XX, Sui WY, Yang JF, Deng YL, Xu J, Huang ZF, Yang JL. Risk factors for neurological complications in severe and rigid spinal deformity correction of 177 cases. BMC Neurol. 2020 Nov 28;20(1):433. doi: 10.1186/s12883-020-02012-8. PMID: 33246421; PMCID: PMC7697368.*	*Does not use multivariable regression to assess risk factors*
6	*Clark AJ, Ziewacz JE, Safaee M, Lau D, Lyon R, Chou D, Weinstein PR, Ames CP, Clark JP 3rd, Mummaneni PV. Intraoperative neuromonitoring with MEPs and prediction of postoperative neurological deficits in patients undergoing surgery for cervical and cervicothoracic myelopathy. Neurosurg Focus. 2013 Jul;35(1):E7. doi: 10.3171/2013.4.FOCUS13121. PMID: 23815252.*	*Does not use multivariable regression to assess risk factors*

7	*De la Garza Ramos R, Goodwin CR, Abu-Bonsrah N, Jain A, Miller EK, Huang N, Kebaish KM, Sponseller PD, Sciubba DM. Patient and operative factors associated with complications following adolescent idiopathic scoliosis surgery: an analysis of 36,335 patients from the Nationwide Inpatient Sample. J Neurosurg Pediatr. 2016 Dec;25(6):730-736. doi: 10.3171/2016.6.PEDS16200. Epub 2016 Aug 26. PMID: 27564784.*	*Ineligible population*
8	*Feng B, Qiu G, Shen J, Zhang J, Tian Y, Li S, Zhao H, Zhao Y. Impact of multimodal intraoperative monitoring during surgery for spine deformity and potential risk factors for neurological monitoring changes. J Spinal Disord Tech. 2012 Jun;25(4):E108-14. doi: 10.1097/BSD.0b013e31824d2a2f. PMID: 22367467.*	*Does not use multivariable regression to assess risk factors*
9	*Ghadirpour R, Nasi D, Iaccarino C, Romano A, Motti L, Sabadini R, Valzania F, Servadei F. Intraoperative neurophysiological monitoring for intradural extramedullary spinal tumors: predictive value and relevance of D-wave amplitude on surgical outcome during a 10-year experience. J Neurosurg Spine. 2018 Nov 9;30(2):259-267. doi: 10.3171/2018.7.SPINE18278. PMID: 30497134.*	*Ineligible study design for Key Question, e.g., case series, modeling (e.g., prediction models, thresholds/ROC, etc.)*
10	*Glennie RA, Ailon T, Yang K, Batke J, Fisher CG, Dvorak MF, Vaccaro AR, Fehlings MG, Arnold P, Harrop JS, Street JT. Incidence, impact, and risk factors of adverse events in thoracic and lumbar spine fractures: an ambispective cohort analysis of 390 patients. Spine J. 2015 Apr 1;15(4):629-37. doi: 10.1016/j.spinee.2014.11.016. Epub 2014 Nov 28. PMID: 25450658.*	*Ineligible study design for Key Question, e.g., case series, modeling (e.g., prediction models, thresholds/ROC, etc.)*
11	*Guest JD, Vanni S, Silbert L. Mild hypothermia, blood loss and complications in elective spinal surgery. Spine J. 2004 Mar-Apr;4(2):130-7. doi: 10.1016/j.spinee.2003.08.027. PMID: 15016389.*	*Does not use multivariable regression to assess risk factors*
12	*Huang ZF, Chen L, Yang JF, Deng YL, Sui WY, Yang JL. Multimodality Intraoperative Neuromonitoring in Severe Thoracic Deformity Posterior Vertebral Column Resection Correction. World Neurosurg. 2019 Jul;127:e416-e426. doi: 10.1016/j.wneu.2019.03.140. Epub 2019 Apr 11. PMID: 30981802.*	*Does not use multivariable regression to assess risk factors*
13	*Kashkoush A, Mehta A, Agarwal N, Nwachuku EL, Fields DP, Alan N, Kanter AS, Okonkwo DO, Hamilton DK, Thirumala PD. Perioperative Neurological Complications Following Anterior Cervical Discectomy and Fusion: Clinical Impact on 317,789 Patients from the National Inpatient Sample. World Neurosurg. 2019*	*Ineligible study design for Key Question, e.g., case series, modeling (e.g., prediction models, thresholds/ROC, etc.), ineligible population*

	Aug;128:e107-e115. doi: 10.1016/j.wneu.2019.04.037. Epub 2019 Apr 10. PMID: 30980979.	
14	*Kato S, Fehlings MG, Lewis SJ, Lenke LG, Shaffrey CI, Cheung KMC, Carreon LY, Dekutoski MB, Schwab FJ, Boachie-Adjei O, Kebaish KM, Ames CP, Qiu Y, Matsuyama Y, Dahl BT, Mehdian H, Pellisé F, Berven SH. An Analysis of the Incidence and Outcomes of Major Versus Minor Neurological Decline After Complex Adult Spinal Deformity Surgery: A Subanalysis of Scoli-RISK-1 Study. Spine (Phila Pa 1976). 2018 Jul 1;43(13):905-912. doi: 10.1097/BRS.0000000000002486. PMID: 29894429.*	*Does not use multivariable regression to assess risk factors*
15	*Kelly MP, Lenke LG, Godzik J, Pellise F, Shaffrey CI, Smith JS, Lewis SJ, Ames CP, Carreon LY, Fehlings MG, Schwab F, Shimer AL. Retrospective analysis underestimates neurological deficits in complex spinal deformity surgery: a Scoli-RISK-1 Study. J Neurosurg Spine. 2017 Jul;27(1):68-73. doi: 10.3171/2016.12.SPINE161068. Epub 2017 May 5. PMID: 28475019.*	*Does not use multivariable regression to assess risk factors*
16	*Kim DH, Zaremski J, Kwon B, Jenis L, Woodard E, Bode R, Banco RJ. Risk factors for false positive transcranial motor evoked potential monitoring alerts during surgical treatment of cervical myelopathy. Spine (Phila Pa 1976). 2007 Dec 15;32(26):3041-6. doi: 10.1097/BRS.0b013e31815d0072. PMID: 18091499.*	*Does not use multivariable regression to assess risk factors*
17	*Lee JY, Hilibrand AS, Lim MR, Zavatsky J, Zeiller S, Schwartz DM, Vaccaro AR, Anderson DG, Albert TJ. Characterization of neurophysiologic alerts during anterior cervical spine surgery. Spine (Phila Pa 1976). 2006 Aug 1;31(17):1916-22. doi: 10.1097/01.brs.0000228724.01795.a2. PMID: 16924208.*	*Does not use multivariable regression to assess risk factors*
18	*Lenke LG, Fehlings MG, Shaffrey CI, Cheung KM, Carreon L, Dekutoski MB, Schwab FJ, Boachie-Adjei O, Kebaish KM, Ames CP, Qiu Y, Matsuyama Y, Dahl BT, Mehdian H, Pellisé-Urquiza F, Lewis SJ, Berven SH. Neurologic Outcomes of Complex Adult Spinal Deformity Surgery: Results of the Prospective, Multicenter Scoli-RISK-1 Study. Spine (Phila Pa 1976). 2016 Feb;41(3):204-12. doi: 10.1097/BRS.0000000000001338. PMID: 26866736.*	*Does not use multivariable regression to assess risk factors*
19	*Lewis ND, Keshen SG, Lenke LG, Zywiel MG, Skaggs DL, Dear TE, Strantzas S, Lewis SJ. The Deformity Angular Ratio: Does It Correlate With High-Risk Cases for Potential Spinal Cord Monitoring Alerts in Pediatric 3-Column Thoracic Spinal*	*Does not use multivariable regression to assess risk factors*

	Deformity Corrective Surgery? Spine (Phila Pa 1976). 2015 Aug 1;40(15):E879-85. doi: 10.1097/BRS.0000000000000984. PMID: 26222664.	
20	*Lewis SJ, Gray R, Holmes LM, Strantzas S, Jhaveri S, Zaarour C, Magana S. Neurophysiological changes in deformity correction of adolescent idiopathic scoliosis with intraoperative skull-femoral traction. Spine (Phila Pa 1976). 2011 Sep 15;36(20):1627-38. doi: 10.1097/BRS.0b013e318216124e. PMID: 21897186.*	*Does not use multivariable regression to assess risk factors*
21	*Montalva-Iborra A, Alcanyis-Alberola M, Grao-Castellote C, Torralba-Collados F, Giner-Pascual M. Risk factors in iatrogenic spinal cord injury. Spinal Cord. 2017 Sep;55(9):818-822. doi: 10.1038/sc.2017.21. Epub 2017 Apr 4. PMID: 28374810.*	*Does not use multivariable regression to assess risk factors*
22	*Park T, Park J, Park YG, Lee J. Intraoperative Neurophysiological Monitoring for Spinal Cord Tumor Surgery: Comparison of Motor and Somatosensory Evoked Potentials According to Tumor Types. Ann Rehabil Med. 2017 Aug;41(4):610-620. doi: 10.5535/arm.2017.41.4.610. Epub 2017 Aug 31. PMID: 28971046; PMCID: PMC5608669.*	*Does not use multivariable regression to assess risk factors*
23	*Rocos B, Strantzas S, Zeller R, Lewis S, Tan T, Lebel D. What is the Optimal Surgical Method for Achieving Correction and Avoiding Neurological Complications in Pediatric High-grade Spondylolisthesis? J Pediatr Orthop. 2021 Mar 1;41(3):e217-e225. doi: 10.1097/BPO.0000000000001707. PMID: 33165266.*	*Does not use multivariable regression to assess risk factors*
24	*Sadashivam S, Abraham M, Kesavapisharady K, Nair SN. Long-term outcome and prognostic factors of intramedullary spinal hemangioblastomas. Neurosurg Rev. 2020 Feb;43(1):169-175. doi: 10.1007/s10143-018-1025-2. Epub 2018 Aug 31. PMID: 30171501.*	*Does not use multivariable regression to assess risk factors*
25	*Saiwai H, Okada S, Hayashida M, Harimaya K, Matsumoto Y, Kawaguchi KI, Kobayakawa K, Maeda T, Ohta H, Shirasawa K, Tsuchiya K, Terada K, Kaji K, Arizono T, Saito T, Fujiwara M, Iwamoto Y, Nakashima Y. Surgery-related predictable risk factors influencing postoperative clinical outcomes for thoracic myelopathy caused by ossification of the posterior longitudinal ligament: a multicenter retrospective study. J Neurosurg Spine. 2019 Dec 27:1-7. doi: 10.3171/2019.10.SPINE19831. Epub ahead of print. PMID: 31881534.*	*Does not use multivariable regression to assess risk factors*
26	*Shlobin NA, Raz E, Shapiro M, Clark JR, Hoffman SC, Shaibani A, Hurley MC, Ansari SA, Jahromi BS, Dahdaleh NS, Potts MB. Spinal neurovascular complications with*	*Does not use multivariable regression to assess*

	anterior thoracolumbar spine surgery: a systematic review and review of thoracolumbar vascular anatomy. Neurosurg Focus. 2020 Sep;49(3):E9. doi: 10.3171/2020.6.FOCUS20373. PMID: 32871559.	*risk factors*
27	*Thuet ED, Padberg AM, Raynor BL, Bridwell KH, Riew KD, Taylor BA, Lenke LG. Increased risk of postoperative neurologic deficit for spinal surgery patients with unobtainable intraoperative evoked potential data. Spine (Phila Pa 1976). 2005 Sep 15;30(18):2094-103. doi: 10.1097/01.brs.0000178845.61747.6a. PMID: 16166902.*	*Does not use multivariable regression to assess risk factors*
28	*Toll BJ, Samdani AF, Janjua MB, Gandhi S, Pahys JM, Hwang SW. Perioperative complications and risk factors in neuromuscular scoliosis surgery. J Neurosurg Pediatr. 2018 Aug;22(2):207-213. doi: 10.3171/2018.2.PEDS17724. Epub 2018 May 11. PMID: 29749884.*	*Does not use multivariable regression to assess risk factors*
29	*Ushirozako H, Yoshida G, Hasegawa T, Yamato Y, Yasuda T, Banno T, Arima H, Oe S, Yamada T, Ide K, Watanabe Y, Kurita T, Matsuyama Y. Characteristics of false-positive alerts on transcranial motor evoked potential monitoring during pediatric scoliosis and adult spinal deformity surgery: an "anesthetic fade" phenomenon. J Neurosurg Spine. 2019 Nov 22:1-9. doi: 10.3171/2019.9.SPINE19814. Epub ahead of print. PMID: 31756712.*	*Does not use multivariable regression to assess risk factors*
30	*Vitale MG, Moore DW, Matsumoto H, Emerson RG, Booker WA, Gomez JA, Gallo EJ, Hyman JE, Roye DP Jr. Risk factors for spinal cord injury during surgery for spinal deformity. J Bone Joint Surg Am. 2010 Jan;92(1):64-71. doi: 10.2106/JBJS.H.01839. PMID: 20048097.*	*Does not use multivariable regression to assess risk factors*
31	*Watanabe T, Kanayama M, Takahata M, Oda I, Suda K, Abe Y, Okumura J, Hojo Y, Iwasaki N. Perioperative complications of spine surgery in patients 80 years of age or older: a multicenter prospective cohort study. J Neurosurg Spine. 2019 Dec 17:1-9. doi: 10.3171/2019.9.SPINE19754. Epub ahead of print. PMID: 31846935.*	*Does not use multivariable regression to assess risk factors*
32	*Wilson TJ, Hamrick F, Alzahrani S, Dibble CF, Koduri S, Pendleton C, Saleh S, Ali ZS, Mahan MA, Midha R, Ray WZ, Yang LJS, Zager EL, Spinner RJ. Analysis of the effect of intraoperative neuromonitoring during resection of benign nerve sheath tumors on gross-total resection and neurological complications. J Neurosurg. 2021 Feb 12:1-10. doi: 10.3171/2020.8.JNS202885. Epub ahead of print. PMID: 33578389.*	*Ineligible study design for Key Question, e.g., case series, modeling (e.g., prediction models, thresholds/ROC, etc.)*

33	*Yoo M, Park YG, Cho YE, Lim CH, Chung SY, Kim D, Park J. Intraoperative evoked potentials in patients with ossification of posterior longitudinal ligament. J Clin Monit Comput. 2022 Feb;36(1):247-258. doi: 10.1007/s10877-020-00646-0. Epub 2021 Feb 6. PMID: 33548015.*	*Does not use multivariable regression to assess risk factors*
